# Epigenetic and Phenotypic Responses to Experimental Climate Change of Native and Invasive *Carpobrotus edulis*

**DOI:** 10.3389/fpls.2022.888391

**Published:** 2022-06-17

**Authors:** Josefina G. Campoy, Mar Sobral, Belén Carro, Margarita Lema, Rodolfo Barreiro, Rubén Retuerto

**Affiliations:** ^1^Departamento de Bioloxía Funcional, Facultade de Bioloxía, Universidade de Santiago de Compostela, Santiago de Compostela, Spain; ^2^Biocost, Facultad de Ciencias and Centro de Investigaciones Científicas Avanzadas (CICA), Universidad de A Coruña, A Coruña, Spain

**Keywords:** Aizoaceae, adaptation, DNA methylation, environmental change, ice plant, invasive species, phenotype, trait

## Abstract

Despite the recent discoveries on how DNA methylation could help plants to adapt to changing environments, the relationship between epigenetics and climate change or invasion in new areas is still poorly known. Here, we investigated, through a field experiment, how the new expected climate scenarios for Southern Europe, i.e., increased temperature and decreased rainfall, might affect global DNA methylation in relation to phenotypic variation in individuals of clonal plant, *Carpobrotus edulis*, from its native (Southern African) and invaded (northwestern Iberian Peninsula) area. Our results showed that changes in temperature and rainfall induced phenotypic but not global DNA methylation differences among plants, and the climatic effects were similar for plants coming from the native or invaded areas. The individuals from the Iberian Peninsula showed higher levels of global methylation than their native counterparts from South Africa. We also observed differences between natives and invasive phenotypes in traits related to the pattern of biomass partitioning and to the strategies for water uptake and use and found an epigenetic contribution to phenotypic changes in some leaf traits, especially on the nitrogen isotopic composition. We conclude that the increased temperature and decreased rainfall projected for Southern Europe during the course of the twenty-first century may foster phenotypic changes in *C. edulis*, possibly endowing this species with a higher ability to successful cope the rapid environmental shifts. The epigenetic and phenotypic divergence that we observed between native and invasive plants suggests an intraspecific functional variation during the process of invasion. This result could indicate that phenotypic plasticity and global DNA methylation are related to the colonization of new habitats. Our findings reinforce the importance of epigenetic plasticity on rapid adaptation of invasive clonal plants.

## Introduction

Invasion of natural areas by exotic species is a major cause of biodiversity loss (Mack et al., 2000; McGeoch et al., 2010), a situation that could be particularly aggravated by the ongoing climate change (Walther et al., 2009; Bellard et al., 2013; IPCC, 2014). It is therefore essential to increase our understanding of the mechanisms that underlie the rapid responses of invasive plants to environmental shifts to efficiently manage current and future invasions.

The mechanisms that have been attributed to the huge success of some clonal plant invaders include an effective ecophysiological adaptation to the new environment and major genomic events, such as hybridization and polyploidization. These processes may lead to different patterns of gene expression re-programming and epigenetic modifications, which, in turn, can contribute to phenotypic novelty and plasticity (Verhoeven and Preite, 2014; Fenollosa et al., 2016; Richards et al., 2017; Mounger et al., 2021). Phenotypic plasticity could be especially advantageous for sessile organisms such as plants in a rapidly changing climate by allowing a genotype to express different phenotypes under the influence of different environments (Bradshaw, 1965; Nicotra et al., 2010; Henn et al., 2018). Likewise, it is well established that genetic variation can interact with the environment to alter phenotype and that this interaction may be determinant for evolutionary changes and adaptation (Goldstein and Ehrenreich, 2021). However, current evidence also supports that, even in the absence of genetic variation, phenotypic variation can be triggered by epigenetic modifications, such as DNA methylation (Bossdorf et al., 2010; Verhoeven et al., 2010). This is because epigenetic changes of the genome can alter gene expression and affect how the genotype translates into the phenotype without changing the DNA sequence (Jablonka and Raz, 2009; Verhoeven et al., 2010; Erdmann and Picard, 2020). These epigenetic changes can be related to a variety of ecologically relevant traits (e.g., Bossdorf et al., 2010) that may be inherited to some extent by future generations (e.g., Zhang et al., 2013; Sobral et al., 2021a, 2021b). Thereby, epigenetic mechanisms can contribute to plant adaptation (Schmid et al., 2018) and may provide an alternative source of phenotypic and functional diversity (e.g., Medrano et al., 2014) which could be especially important for clonal plants and plant invaders with low genetic diversity (Verhoeven and Preite, 2014; Mounger et al., 2021).

Furthermore, epigenetic variation may be altered by ecological interactions, providing thereby an additional pathway for evolutionary change (Bossdorf et al., 2008). Plants can alter their epigenetic marks to adjust their responses to abiotic factors such as temperature, drought, salt and nutrient stress (Verhoeven et al., 2010; Nicotra et al., 2015; González et al., 2017), elevated atmospheric CO_2_ concentration (Saban et al., 2020), or heavy metals (Shafiq et al., 2019). It has also been demonstrated that epigenetic mechanisms may be relevant for biotic interactions (see Alonso et al., 2019 for a review), for adaptation to different environments and habitat conditions (Lira-Medeiros et al., 2010; Richards et al., 2012; Foust et al., 2016), for phenotypic variation between introduced and native populations (Banerjee et al., 2019, and references therein), and for helping plants to colonize new habitats (Zhang et al., 2016; Liu et al., 2018). Thereby, because epigenetic variation may contribute to fast plant adaptation to novel environments, this process may be an important mechanism for invasive success. However, despite these recent discoveries on epigenetic regulation in plants, the relationship between epigenetics and responses to climate change or invasion of new areas is still poorly known. Progress on this matter requires research combining the study of plant epigenetic variation and their ecologically relevant phenotypic effects over a range of environmental conditions and regions of origin.

Alien invasive species, such as *Carpobrotus edulis* (L.) N.E. Br., native to the South African, often face sudden environmental changes when they colonize new territories. This offers a good opportunity to examine how these species cope with novel environments and whether this process influences the phenotype and/or the epigenotype. In this study, we used a set of ecophysiological measurements and methylation-sensitive amplified fragment length polymorphism markers (MSAP) to assess how the increased temperature and decreased rainfall predicted for Southern Europe affect global DNA methylation in relation to the phenotypic variation of *C. edulis*. To this end, and considering the temperature and rainfall projections for Southern Europe over the twenty-first century (IPCC, 2014; EEA, 2017), we studied the responses of *C. edulis* individuals from the native (South Africa) and the invaded region (northwestern Iberian Peninsula) to simulated climatic scenarios in a field experiment.

Specifically, in this work we addressed two questions: (1) might the increased temperature and decreased rainfall predicted for Southern Europe induce phenotypic and epigenetic changes in *C. edulis?* In a previous research we showed that the traits considered in this study have functional consequences for the responses of *C. edulis* to climate change (Campoy et al., 2021). Moreover, because epigenetic regulation may be an important source of phenotypic plasticity and thus can contribute to increase the existing phenotypic and functional biodiversity, we expect that the new climate scenarios will foster rapid phenotypic and epigenetic changes in this species. (2) Are there phenotypic and epigenetic differences among *C. edulis* from the native and invaded areas*?* Considering the potential role of epigenetics in plant invasion (Mounger et al., 2021), we expect high phenotypic and epigenetic plasticity in individuals from the invaded regions.

## Materials and Methods

### Study Plant

*Carpobrotus edulis* (L.) N.E. Br. (Aizoaceae) is a succulent perennial plant native from Southern Africa (Wisura and Glen, 1993). It was introduced in the five worldwide Mediterranean-type ecosystems more than 100 years ago, and since then, this species has become invasive in many coastal habitats (review by Campoy et al., 2018). The combination of sexual and asexual (clonal) reproduction in *C. edulis*, together with the high hybridization potential of the species (Vilà and D’Antonio, 1998; Suehs et al., 2004), explains its worldwide successful propagation. The high plant invasiveness under a wide range of stressful conditions (e.g., drought, warming, or strong irradiance) has also been related to a high morphological and ecophysiological plasticity in growth, biochemical, and physiological traits (Campoy et al., 2017, 2021; Fenollosa et al., 2017) and to the ability of the species to use and modify soil resources (e.g., Fenollosa et al., 2016; Vieites-Blanco and González-Prieto, 2018).

### Sampled Sites and Experimental Design

The experimental plant material was sampled between January and April 2015 from eight sites, four from the native area (Cape Region, South Africa) and four from the invaded area (northwest of the Iberian Peninsula, Southern Europe; Supplementary Table 1). To have a more comprehensive illustration of the variability in each region, we collected, within each site, plants separated at least 25 m from the others. After collection, plants were transported in polystyrene trays from the original sites to the glasshouse of the University of Santiago. Then, plants were transplanted into individual 2.5-L pots filled with dune sand until the beginning of the experiment. To prevent hydric stress, plants were watered according to their requirements (approximately once or twice per week).

The study was conducted on a permanent field plot (21 m long × 12 m wide) located over a secondary dune (IGME, 2014) on the island of Sálvora (42°28′44″N, 9°0′34″W; northwest Iberian Peninsula; Supplementary Figure 1). Inside this plot, we experimentally manipulated the climate in 32 subplots of 1.27 m^2^ to establish a full factorial experimental design with three factors: region of plant origin (native vs. invaded), temperature (control vs. increased), and rainfall (control vs. reduced). To modify temperature conditions, we installed methacrylate open top chambers (OTCs) as those typically employed in warming experiments (e.g., Hollister and Webber, 2000; Yahdjian and Sala, 2002; Maestre et al., 2013), in eight of the subplots. Throughout the study period (September 2015–November 2016), the OTCs increased the air temperature by 2.0°C, on average (Campoy et al., 2021), a realistic scenario for the study area according to the predictions of the PROMES regional atmospheric model (Castro et al., 1993). To achieved the rainfall reduction predicted in the study area (~33%), we placed methacrylate rainfall collectors as those commonly used in manipulative climatic experiments (e.g., Yahdjian and Sala, 2002), in another eight subplots. To examine the combined effects of increased temperature and reduced rainfall, we installed an OTC below the rainfall collectors in eight other subplots. Finally, the eight remaining subplots were not modified to represent the current climatic conditions (C). The island’s climate is “Mediterranean sub-humid of Atlantic tendency” (Allué, 1966) or “Warm temperate, with dry and warm summers” (*Csb*), under the Köppen−Geiger climate classification (Kottek et al., 2006). The mean annual rainfall is 1,193 L/m^2^ and the mean temperature of the warmest (August) and the coldest (December) month is 20°C and 10°C, respectively.^1^ For a more detailed information of experimental chambers and the different sensors used to monitor the climatic conditions in the field plot (see Campoy et al., 2021).

In September 2015, we carefully removed the natural vegetation growing in the 32 subplots, where we transplanted into the soil a total of 64 plants, eight from each of the sites of the invaded and the native area. Specifically, we haphazardly assigned two plants to each subplot: one from the invaded area and one from the native area. Thus, 8 plants per region (two per site) were grown under each of the four climatic treatment (eight subplots per treatment). Because we lost two replicates during the progress of the experiment due to non-demonic intrusion (*sensu* Hurlbert, 1984), the number of individuals considered in the final design was reduced to 48 plants (24 from the native and 24 from the invaded regions), i.e., six replicates for each treatment combination. These replicates came from the four sites in their respective region, so all sites were included in our final design, although they were not equally represented.

### Phenotypic Traits

To provide an estimate of the radiation use efficiency and the photosynthetic pigment contents of leaves, we measured the reflectance spectra (from 300 to 1,000 nm) in all plants at intervals of ~2 months (between November 2015 and November 2016), with a portable spectrometer (Unispec, PP Systems Haverhill, MA, United States). Reflectance data were processed using AVICOL v.6 software (Gomez, 2006), and the structural independent pigment index [SIPI = (*R*_800_ − *R*_445_)/(*R*_800_ − *R*_680_)] and the photochemical reflectance index [PRI_531_ = (*R*_531_ − *R*_570_)/(*R*_570_ + *R*_531_)] were calculated. The former provides a semi-empirical estimation of the carotenoid-to chlorophyll *a* ratio (Peñuelas et al., 1995b), and the latter is directly correlated with radiation use efficiency (RUE, mol CO_2_∙ mol^−1^ photons; Gamon et al., 1992; Peñuelas et al., 1995a) and inversely correlated with the dissipation of excess radiation energy as heat (Guo and Trotter, 2004). Due to the repeated measure nature of leaf reflectance data (SIPI and PRI indices), for this study we calculated the mean values of the eight set of measurements performed throughout the 14 months of the duration of the experiment.

To determine the proportion of biomass allocated to roots, we used the root-to-shoot ratio (RSR), calculated as RSR = root dry mass/shoot dry mass. To assess plant growth under the experimental conditions, we calculated the relative growth rate (RGR) as follows, RGR = [ln (dry weight *t_2_*) − ln (dry weight *t_1_*)]/(*t*_2_ − *t*_1_), where *t*_2_ and *t*_1_ are the final time and the initial time, respectively (Villar et al., 2004).

For determination of C and N percentages of dry mass (C/N ratio) and the molar ^15^N/^14^N (δ^15^N) and ^13^C/^12^C (δ^13^C) ratios, a composite sample of leaves (~2–3 mg dry wt.) from the three apical-most ramets in each plant was ground in a ball mill (Mixer Mill 400 Retsch GMBH, Haan, Germany) and then analyzed at the Research Support Services of the University of A Coruña (Spain). Carbon and N isotope ratios were expressed relative to the composition of a standard (Pee Dee belemnite [PDB] CaCO_3_ for C, and atmospheric N for N). The δ values (‰) were calculated as [(*R_sam_/R_std_*) − 1] × 1,000, where *R* refers to the ^13^C/^12^C or ^15^N/^14^N ratio in the plant sample and standard, respectively. Polyethylene (International Atomic Energy Agency [IAEA C6]) and (NH_4_)_2_SO_4_ (IAEA N1) were used as secondary international isotope standards for C and N, respectively. The δ^13^C values were transformed into ∆^13^C values by using the following expression: ∆^13^C = (δ^13^C_air_ − δ^13^C_plant_)/(1 + δ^13^C_plant_), assuming a δ^13^C air value of −8‰ on the PDB scale (Farquhar et al., 1989). Plant ^13^C/^12^C and the ^15^N/^14^N isotopic signatures provide valuable and integrated information on the water use efficiency (WUE), and the source, absorption, and assimilation of nitrogen, respectively (Robinson, 2001; Dawson et al., 2002).

### Epigenetic Analyses

For epigenetic analyses and for testing its reproducibility, we collected two opposite, fully developed, and healthy leaves from each plant (*n* = 48). To guarantee that all leaves had a similar development stage, samples were taken from the apical-most ramets of the main shoots (stolons).

#### DNA Extraction and MSAP Reactions

From each plant, we extracted the total genomic DNA from 20 mg fresh leaf tissue using the Maxwell® 16 LEV Plant DNA extraction Kit (PROMEGA) following manufacturer instructions. To avoid cross-contamination, each leaf was dissected using disposable tools and/or flame-sterilized material. The integrity of extracted DNA was verified by electrophoresis on 1.5% agarose gels. After DNA quantification using a Tecan Genios Microplate reader and the Quant-iT dsDNA Assay Kit, High-Sensitivity (HS; Thermo Fisher Scientific), samples were normalized to 5 ng·μl^−1^ and stored at −20°C.

The methylation-sensitive amplified polymorphism (MSAP) reactions were performed following the general steps described by (Vos et al., 1995) for the Amplified Fragment Length Polymorphism (AFLP), with some modifications. We used the same *Eco*RI endonuclease as rare cutter and replaced the frequent cutter *Mse*I in two parallel runs by the methylation-sensitive restriction enzymes *Hpa*II and *Msp*I. The two isoschizomers recognize and cleave the same tetranucleotide sequence 5-CCGG but differ in their sensitivity to the methylation state of cytosine (Pérez-Figueroa, 2013; Schulz et al., 2013).

Briefly, the MSAP protocol was performed as follows. For each sample, 10 μl of genomic DNA (*ca.* 50 ng) was restricted in a total volume of 20 μl containing 10X CutSmart Buffer (BioLabs), 2.5 units of *EcoRI* (BioLabs), and 2.5 units of *Hpa*II or *Msp*I (BioLabs) for 3 h at 37°C and 20 min at 70°C. After incubation, 20 μl of digested fragments was added, in parallel reactions, to 6 μl of a ligation solution containing 0.1 μM *EcoRI*-adapters (Eurofins MWG Operon), and 1 μM *Hpa*II*/Msp*I-adapters (Macrogen; see Supplementary Table 2 for adaptor sequences), 0.52 units of T4 DNA ligase (Fermentas) and 10X ligation buffer (Fermentas). The ligation was carried out for 3 h at 37°C. Ligation products were diluted 10-fold in Milli-Q H_2_O (Millipore Co.), and 10 μl was used for a pre-selective amplification with 0.3 μM *EcoRI*-primer (Macrogen), 0.3 μM *HpaII/MspI*-primers (Macrogen; see Supplementary Table 2 for primer sequences), 2.5 mM MgCl_2_, 10X PCR buffer, 0.04 μgμl^−1^ BSA, 0.2 μM dNTPs, and 0.4 U of *AmpliTaq Gold* polymerase (Applied Biosystems) in a final volume of 20 μl. The thermocycler protocol was 2 min at 72°C, 2 min at 94°C, 20 cycles of 30 s at 94°C, 30 s at 56°C, and 2 min at 72°C, followed by a final extension of 30 min at 60°C. Two microliters from the 8-fold diluted products of the pre-amplification was finally used for selective amplifications with 0.6 μM of each *Eco*RI, *Hpa*II*/Msp*I selective primers (Macrogen), 0.8 μM dNTPs, 2.5 mM MgCl_2_, 0.04μgμl^−1^ BSA, 10X PCR buffer, and 0.4 units of *HotStartTaq Gold* polymerase (Applied Biosystems) in a final volume of 10 μl. Thermocycler conditions for selective amplification were: 4 min at 95°C, 12 cycles of 30 s at 94°C, 30 s at 65°C (first cycle, then decreasing 0.7°C for each one of the last 11 cycles), and 2 min at 72°C; 26 cycles of 30 s at 94°C, 30 s at 56°C, and 2 min at 72°C, followed by a final extension of 30 min at 72°C. Preamplification was performed immediately after ligation, whereas the products of the other reactions were kept overnight at −20°C. PCRs were performed in a Hybaid thermocycler model PxE (Thermo Fisher Scientific Inc., Waltham, MA, United States). Reactants were always mixed in a laminar flow cabin; DNA and PCR product solutions were always added using filter tips to minimize the risk of cross-contamination.

After an initial screening of 28 primer combinations, we choose six selective primer combinations (1: *Eco*RI + AA/ *Hpa*II*/Msp*I *+* AC; 2: *Eco*RI + AT/ *Hpa*II*/Msp*I *+* AA; 3: *Eco*RI + AT/ *Hpa*II*/Msp*I *+* AC; 4: *Eco*RI + TA/ *Hpa*II*/Msp*I *+* AA; 5. *Eco*RI + TC/ *Hpa*II*/Msp*I *+* AT; 6. *Eco*RI + TG/*Hpa*II*/Msp*I *+* AC; see Supplementary Table 3 for sequences). The 5′ end of the selective *E*-primers was labeled with FAM, HEX, or NED fluorochromes. PCR fragments were separated on an ABI 3130xl Genetic Analyzer (Applied Biosystems Foster City, United States) with automated DNA (Applied Biosystems) sequencer with HD-500 as size standard (Applied Biosystems). The selected paired primer combinations for MSAP analyses yielding the highest levels of polymorphism were tested again on new, independent DNA extractions of 48 replicate samples (100%) to assess reproducibility. The overall scoring error rate (3.89%; see also Supplementary Table 3 for scoring error rate by primer combination) was consistent with former studies (e.g., Herrera and Bazaga, 2010).

Fingerprint patterns of MSAP profiles were processed with the software GeneMarker v1.70 (Softgenetics LLC, State College, PA, United States). The scoring was blindly done by the same person (JGC) following common recommendations for AFLP markers (Whitlock et al., 2008), with minor modifications. Peak height data were exported and for each fragment a specific peak height threshold was manually determined based on the peak height distribution which allowed scoring presence (1) and absence (0) of the fragments.

#### MSAP Scoring

To determine the DNA methylation status of every locus from the presence/absence scores of both *Eco*RI-*Msp*I and *Hpa*II*/Msp*I reactions, we used the R script *MSAP_calc* provided by (Schulz et al., 2013), following the methylation transformation scheme described by (Herrera and Bazaga, 2010). Under this approach, for every individual and particular fragment, it was first determined whether the fragment was: (1) present in both *Eco*RI*/Hpa*II and *Eco*RI*/Msp*I profiles, indicating a non-methylated state (condition I, pattern 1/1); (2) present only in either *Eco*RI*/Msp*I or in *Eco*RI*/Hpa*II profiles, denoting, respectively, a methylated state of ^HMe^CG- or ^Me^CG-sites (condition II, pattern 0/1) or a methylated state of ^HMe^CCG-sites (condition III, pattern 1/0); and (3) absent from both *Eco*RI*/Msp*I profiles (condition IV, pattern 0/0), representing an uninformative state as it can have multiple and equivocal reasons (Pérez-Figueroa, 2013; Schulz et al., 2013). Methylation-susceptible fragments were then scored as 0, for the non-methylated state (condition I); 1, for the methylated state (conditions II and III); and unknown (i.e., score missing) for uninformative condition IV (Herrera and Bazaga, 2010; Morán and Pérez-Figueroa, 2011; Schulz et al., 2013). For the purpose of this study, we only used the “methylation-susceptible loci” obtained with a fixed threshold of 5% as performed by (Schulz et al., 2013). Based on the selecting scoring strategy (Herrera and Bazaga, 2010), we obtained a total of 223 fragments (Supplementary Table 3), from which we calculated the percentage of global DNA methylation.

### Statistical Analyses

The effect of treatments on global DNA methylation was analyzed using a linear mixed model (LMM) with region of origin (native vs. invaded), temperature (control vs. increased temperature), and rainfall (control vs. reduced rainfall) as fixed factors, and the sampling sites of the populations as a random effect nested within region of origin.

To analyze the effect of treatments and global DNA methylation on the phenotype of *C. edulis* plants from both native and invaded regions, we first summarized the seven selected phenotypic traits (RGR, RSR, C/N, Δ^13^C, δ^15^N, SIPI, and PRI_531_ indices) using principal component analysis. The selection of phenotypic traits was based on previous findings on the ecophysiological responses of native and invasive genotypes of *C. edulis* to climate change (Campoy et al., 2021). The first three principal components, jointly explaining approx. 70% of the variation, were included in the following analyses as response variables in three different LMM models to test the effects of region, temperature, rainfall, and global DNA methylation, on these three principal components. Site nested within region was also included as a random effect in these models. We performed Pearson correlations to assess to which extent each principal component was linearly related to global DNA methylation. All variables fitted a normal distribution, and no transformations were required. LMM parameters were estimated using a restricted maximum likelihood (REML) approach. To examine the effects of treatments on variables, *p*-values were estimated using Satterthwaite degrees of freedom. Following the AICc criterion, interaction terms were included or excluded in the models when appropriate (Burnham and Anderson, 2002). Statistical analyses were made in IBM SPSS Statistics v25. A *p*-value ≤ 0.05 was considered as statistically significant.

Finally, to analyze the simultaneous relationships of region of plant origin, rainfall, and temperature, with global DNA methylation and the phenotype (represented by the three principal components of the PCA), we performed structural equation models (SEM). For the SEM analyses, all variables were standardized, and tested models were based on our previous knowledge of the species (Campoy et al., 2021). Different models (see Supplementary Figures 2 3 for some examples) were analyzed. Model selection was made by testing the goodness of fit of the models using the means of maximum likelihood estimation on the variance–covariance matrix. A non-significant goodness of fit test indicates that the model is a good description of the observed covariance among the variables (Grace, 2006). Structural equation modeling was performed with SEPATH procedure in STATISTICA (StatSoft Inc., 2011).

## Results

### Differences in Methylation Between *Carpobrotus edulis* From the Native and Invaded Areas

**Figure 1:**
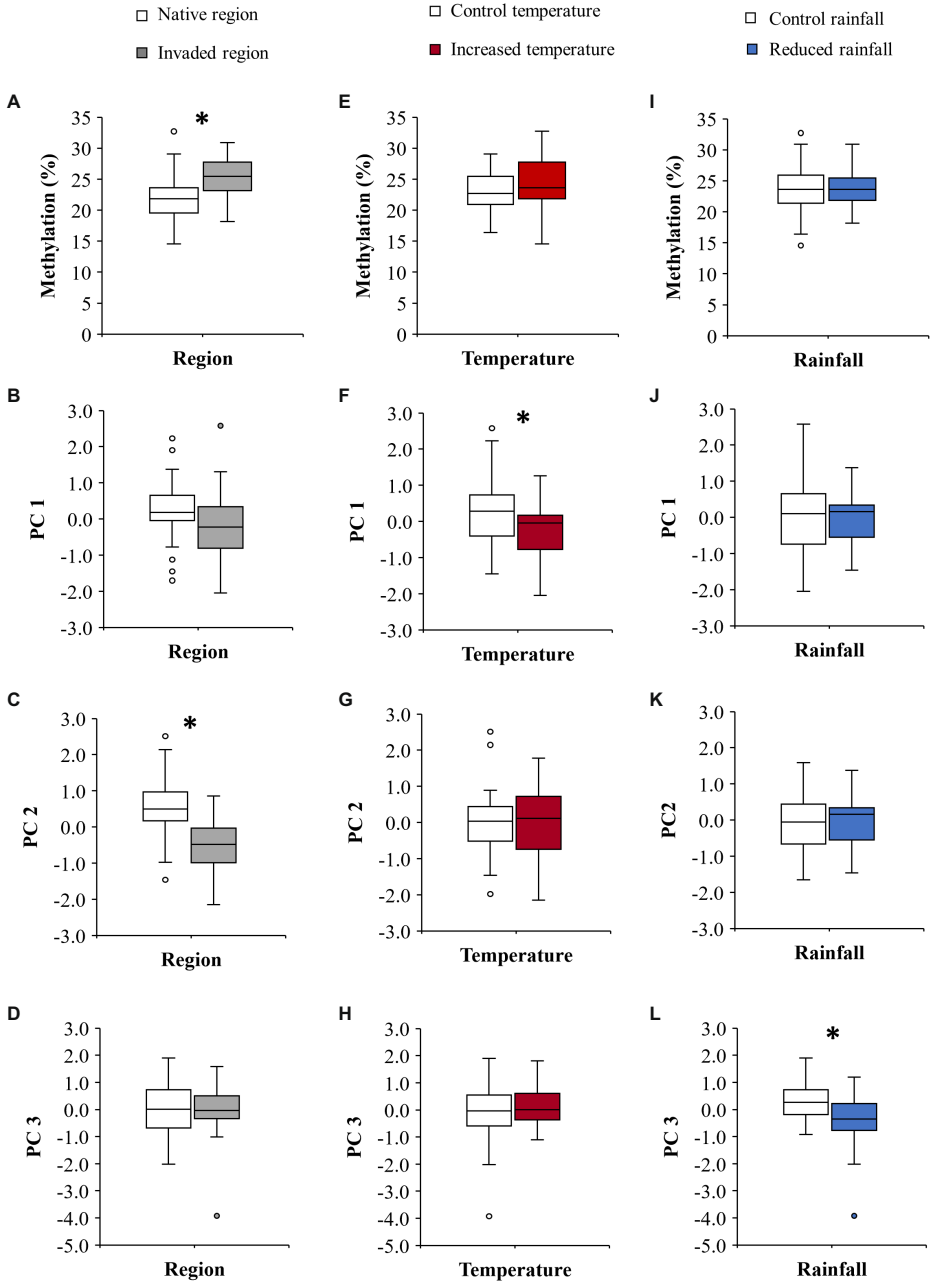
Effect of region (native vs. invaded) on the global DNA methylation **(A)**, the first principal component (PC 1 = 35.1% variance; **B**), the second principal component (PC 2 = 21.2% variance; **C**), and the third principal component (PC 3 = 14.5% variance; **D**) of *C. edulis* phenotype. **(E–H)** show the effect of temperature (control vs. increased) on global methylation **(E)**, PC 1 **(F)**, PC 2 **(G)**, and PC 3 **(H)**. **(I–L)** show the effect of rainfall (control vs. reduced) on global methylation **(I)**, PC 1**(J)**, PC 2 **(K)**, and PC 3 **(L)**. The boxplots show the median, interquartile range, minimum, maximum, and the outliers; *n* = 24. Significant differences are denoted by *. See text for statistics.

The *C. edulis* plants from the native and the invaded region differed in their global DNA methylation, which was significantly higher in plants from the invaded region (Table 1; Figure 1A). We did not detect any significant effect of temperature or rainfall treatments on the global DNA methylation (Table 1; Figures 1E,I).

**Table 1:** Results of linear mixed model examining the effect of region, temperature, and rainfall on the global DNA methylation of *Carpobrotus edulis* plants.

Methylation (%)						
Effects	Variance	SE	Estimate	df	*F*	*p*
**Nested random**						
Site(region)	0.370	1.708				
**Fixed**						
Region			−3.061	1, 6	6.496	**0.044**
Temperature			−1.359	1, 40	1.474	0.232
Rainfall			−0.276	1, 40	0.061	0.807

*Site nested within region was included as a random effect in the model (estimate of residual ± SE = 14.992 ± 3.417). Values of *p* < 0.05 are highlighted in bold. See Figure 1 for significant differences for main effects (*n* = 48)*.

### Phenotypic Variation of *Carpobrotus edulis* in Function of Plant Origin, Climatic Treatment, and Global DNA Methylation

Seventy percent of the total variation of the seven phenotypic traits measured on *C. edulis* plants was explained by the first three principal components, which accounted for 35.1%, 21.2%, and 14.5% of the total variance, respectively (Supplementary Table 4).

**Figure 2:**
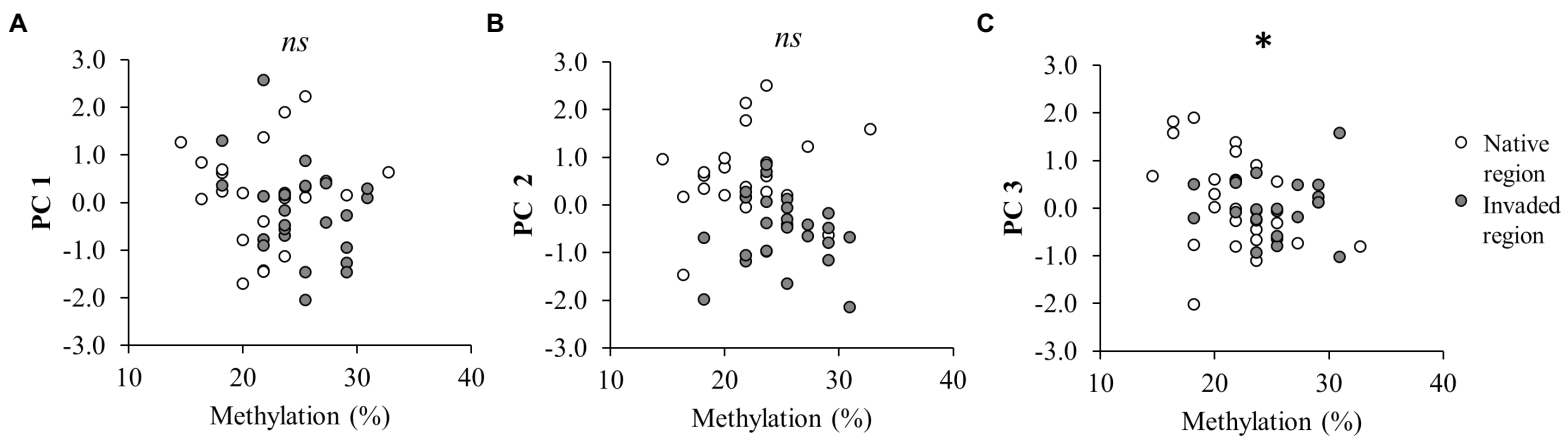
Effect of global DNA methylation on the first principal component (PC 1 = 35.1% variance; *F*_1,37_ = 0.95; *p* = 0.337; **A**), the second principal component (PC 2 = 21.2% variance; *F*_1,38_ = 0.32; *p* = 0.575; **B**), and the third principal component (PC 3 = 14.5% variance; F_1,38.9_ = 6.73; *p* = 0.013; **C**) of *C. edulis* phenotype (*n* = 48). Significant correlation between methylation and principal components is denoted by *, whereas non-significant correlation is denoted by *ns*.

Results of LMM showed that temperature significantly decreased the PC 1 values (Table 2; Figure 1F). We did not detect significant effects of rainfall on PC1 (Table 2; Figure 1J). The PC 2 was the only component that was different between regions of origin (Table 2; Figures 1B–D), with plants from the invaded region showing lower values than plants from the native region (Figure 1C). We did not detect significant effects of temperature or rainfall on PC 2 (Table 2; Figures 1G,K). LMM also showed that reduced rainfall significantly decreased the PC 3 values of plants (Table 2; Figure 1L), but the magnitude of this effect marginally depended on temperature (Table 2; Supplementary Figure 4). This result means that the effect of reduced rainfall in decreasing δ^15^N was only manifested in plants under the control temperature (Supplementary Figure 4C). Interestingly, only the PC 3 was significantly correlated with methylation (Pearson’s *r* = −0.312; *p* = 0.031; Table 2; Figure 2), showing lower values with increasing values of methylation (Figure 2C).

**Table 2:** Results of linear mixed models examining the effects of region (Re), temperature (T), rainfall (R), and genome global methylation, on the three principal components (PC 1, PC 2, PC 3) of the PCA performed on seven phenotypic traits (RGR, RSR, C/N, Δ^13^C, δ^15^N, SIPI, and PRI_531_ indices) of *Carpobrotus edulis.*

Effects	PC 1 (=35.1% variance)						PC 2 (=21.2% variance)						PC 3 (=14.5% variance)					
	Variance	SE	Estimate	df	*F*	*P*	Variance	s.e	Estimate	df	*F*	*P*	Variance	s.e	Estimate	df	*F*	*P*
**Nested random**																		
Site(region)	0.258	0.222					0.070	0.124					0.001	0.091				
**Fixed**																		
Region			−0.065	1, 7	0.169	0.694			1.392	1, 6	12.009	**0.014**			−0.843	1, 5	0.001	0.979
Temperature			0.137	1, 34	4.560	**0.040**			0.451	1, 34	0.124	0.727			−1.242	1, 34	1.156	0.290
Rainfall			−0.523	1, 34	0.267	0.609			0.375	1, 33	0.515	0.478			−0.289	1, 34	9.751	**0.004**
Methylation			−0.035	1, 37	0.947	0.337			0.019	1, 38	0.320	0.575			−0.087	1, 39	6.729	**0.013**
Re x T			0.096	1, 34	0.018	0.895			0.070	1, 33	0.282	0.599			0.964	1, 34	1.131	0.295
Re x R			0.573	1, 34	0.635	0.431			−0.296	1, 34	1.649	0.208			1.146	1, 34	2.027	0.164
T x R			0.900	1, 35	2.042	0.162			−0.473	1, 35	2.717	0.108			1.417	1, 36	3.892	*0.056*
Re x T x R			−0.327	1, 34	0.101	0.752			−0.651	1, 35	0.454	0.505			−0.875	1, 36	0.781	0.383

*Site nested within region was included as a random effect in the models (estimates of residuals ± SE = 0.766 ± 0.187 for the PC 1, 0.687 ± 0.171 for the PC 2, and 0.734 ± 0.183 for the PC 3). Values of *p* < 0.05 are highlighted in bold, and values of *p* marginally significant are highlighted in italics. See Figure 1 and Supplementary Figure 4, for significant differences*.

### Integrating the Relationships Between Phenotypic and Epigenetic Variation of *Carpobrotus edulis*

**Figure 3:**
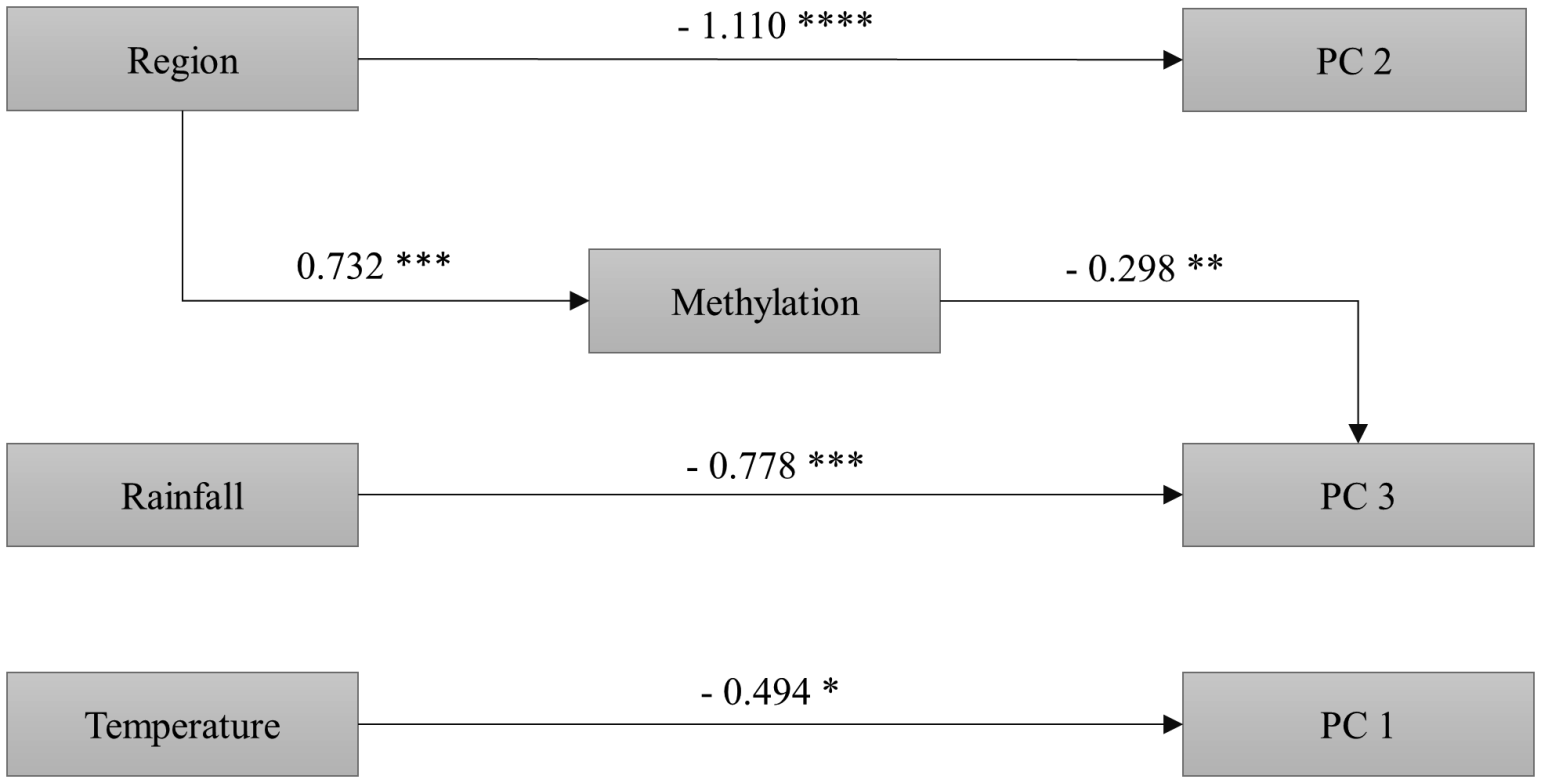
Results of the structural equation model analyzing the relationship of region of plant origin (native and invaded region), rainfall (control and reduced rainfall), and temperature (control and increased temperature), with global DNA methylation and the three principal components of the PCA performed on seven phenotypic traits (RGR, RSR, C/N, Δ^13^C, δ^15^N, SIPI, and PRI_531_ indices) of the invasive plant *C. edulis*. We show coefficients for each significant path. *p < 0.1, **p < 0.05, ***p < 0.01, ****p < 0.001. *N* = 48 plants. Model properly fitted the data as shown by the non-significant Chi-squares values (χ^2^: 4.015; df: 12; *p*: 0.983). Initial model and other alternative models can be found in **Supplementary Figures 2**, **3**.

As shown by the SEM, neither temperature nor rainfall significantly influenced the epigenotype (global DNA methylation), but the epigenotype was different in function of plant origin (higher global methylation in plants from the invaded region; Figure 3).

Region of origin was not associated with PC1 (PRI_531_ and SIPI indices, RGR, and C/N), but it explained variation in PC 2 (i.e., on the Δ^13^C and RSR; Figure 3). Also, our results showed that region of origin indirectly influences PC 3 (mainly the δ^15^N), through its effect on methylation (Figure 3). The PC 3 was the only principal component affected by methylation and by rainfall (Figure 3). Finally, the SEM showed that PC 1 variables were affected by temperature (Figure 3; see also Supplementary Figures 2 3).

## Discussion

Current and future global change scenarios challenge the capacity of plants to respond to the rapid shifts in environmental conditions. By testing the connection between epigenetic mechanisms and phenotypic responses in native and invasive populations of *C. edulis*, this study showed that plants can adapt to shifts in environmental conditions through phenotypic plasticity. In addition, environmental-induced epigenetic modifications can contribute to extending phenotypic and functional diversity, which may also help plants to cope with these changing environments and to colonize new habitats.

### Climatic Effects on Epigenetic and Phenotypic Responses and Relationship Between DNA Methylation and Phenotypes

Our findings support our hypothesis that the new climate scenarios predicted for Southern Europe may foster rapid phenotypic changes in *C. edulis,* but, contrary to our predictions, we did not detect any significant variation in global DNA methylation in response to experimental climate change.

The increase in temperature primarily affected phenotypic traits (PC 1 = 35.1% of the total variance) of *C. edulis* related to some relevant physiological and plant growth characteristic (Table 2; Supplementary Table 4). Specifically, loadings for traits associated with this principal component (RGR, PRI_531_ and SIPI indices, and C/N) indicate that higher temperatures increased the relative growth rate and the photochemical efficiency of plants whereas decreased the proportion of carotenoids to chlorophyll *a* and the ratio of carbon to nitrogen. Previous studies have demonstrated that PRI is inversely correlated with the dissipation of excess radiation energy as heat, and with the SIPI index (i.e., with the ratio of carotenoids to chlorophylls *a;* Peñuelas et al., 1995b; Guo and Trotter, 2004), and directly correlated with the net CO_2_ uptake and photosynthetic radiation-use efficiency (RUE, mol CO_2_∙ mol^−1^ photons; Gamon et al., 1992; Peñuelas et al., 1995a). The observed higher values of the PRI index and lower values of the SIPI index at elevated temperature therefore suggest that native and invasive plants can protect photosynthesis from high temperatures with a low investment in photoprotection, thus allowing plants to obtain a high photochemical efficiency and, consequently, a high RGR. These results are consistent with findings of our previous research in which we showed that the highly plastic response of *C. edulis* to global warming in terms of growth, physiology, and biochemistry may increase the expansion of the species under warmer climates (Campoy et al., 2021). These outcomes also reinforce current evidence that high plasticity levels may enable plants to better tolerate the rapid shifts in environmental conditions and the idea that plasticity could play an important role in invasions (Richards et al., 2006; Nicotra et al., 2015; Henn et al., 2018).

Regarding foliar chemistry and stoichiometry of plants, in a recent meta-analysis of experimental field studies, (Xu et al., 2020) examined shifts in foliar ratios in response to several global change drivers, and their results showed that variation in foliar C/N was mostly explained by shifts in atmospheric nitrogen deposition (i.e., N addition) but not by experimental warming. This result contrasts somewhat with our observation that increased temperature decreased leaf C/N ratio of plants. This change in the C/N ratio could be related to an increase leaf transpiration rate under warming conditions. A higher transpiration rate would imply higher water requirement and greater nutrient translocation from the soil to the plant, which could lead to an increase in N concentration and, consequently, a decrease in the C/N ratio (Jauregui et al., 2015; Wang et al., 2019). Because the leaf C/N ratio plays an important function in ecosystem energy and nutrient dynamics (Zhou et al., 2019; Xu et al., 2020), our findings suggest that the observed shift in this foliar trait of *C. edulis* in response to warming may have a large impact upon ecosystem function at invaded sites.

Our study also revealed that the effect of reduced rainfall in decreasing PC 3 values under the control temperature, may affect phenotypic aspects of *C. edulis* (PC 3 = 14.5% of the total variance; Table 2; Supplementary Figure 2C) mainly related to the ^14^N:^15^N isotope ratio of plant tissues (i.e., δ^15^N; Supplementary Table 4). (Campoy et al., 2021) demonstrated a similar relationship between temperature and water availability and leaf δ^15^N in *C. edulis*, but these findings contrast with the commonly reported patterns showing that plant δ^15^N usually decreases with increasing mean annual precipitation and with decreasing mean annual temperature (Amundson et al., 2003). Nitrogen isotopes have been widely applied in ecological studies because plant variation in δ^15^N is strongly associated with many important biogeochemical processes including N mineralization, ammonia volatilization, nitrification, and denitrification (Makarov, 2009; Chen et al., 2018). However, comparison across studies and the identification of the precise N processes that can be affected are highly complex because the variation in δ^15^N in plants can be determined by the combined effect of several interrelated factors (Makarov, 2009; Wang and Liu, 2011; Chen et al., 2018; Henn et al., 2018). In fact, another remarkable finding of our research is that the variation of the PC 3, and thus δ^15^N, was not only significantly correlated with climatic treatments, but also with global DNA methylation, showing lower values with increasing values of methylation (Table 2; Figure 2C). These results would indicate that epigenotype and climatic treatments did not affect the whole phenotype of *C. edulis* but affected specific trait combinations.

The contribution of DNA methylation to phenotypes has been previously documented using both model and non-model species (review in Richards et al., 2017). For instance, Bossdorf et al. (2010) used *Arabidopsis thaliana* (L.) Heynh to demonstrate that experimental alteration of DNA methylation can cause major shifts in plant phenotypes affecting not only means and variability of growth, fitness, and phenological traits, but also their phenotypic plasticity. Likewise, Zhang et al. (2013) provided evidence of heritable variation among epigenetic recombinant inbred lines of *A. thaliana* in root allocation and in the plasticity to drought and nutrient levels. Other studies using wild plant populations of some species such as *Ilex aquifolium* L., *Viola cazorlensis* Gand., and *Helleborus foetidus* L. also found correlations between anonymous MSAP markers and ecologically important leaf traits (Herrera and Bazaga, 2013), flower morphology (Herrera and Bazaga, 2010), and fitness-related traits (Medrano et al., 2014).

Furthermore, several studies have documented changes in DNA methylation with exposure to different environmental stresses (Verhoeven et al., 2010; Nicotra et al., 2015; González et al., 2017; Shafiq et al., 2019; Saban et al., 2020), which can be transgenerational (Verhoeven et al., 2010; Sobral et al., 2021a). However, in our experiment, we did not detect any significant changes in global DNA methylation between control plants and plants grown under the experimental climatic conditions for 14 months. This interesting finding provides evidence that DNA methylation at the whole-genome level in *C. edulis* does not vary in response to increase in temperature, reduction in rainfall, or by the combined effects of both stressful climatic factors, suggesting that the observed phenotypic variation in response to our experimental climatic conditions was not related to changes in the epigenotype of the species. The high plasticity for morphological and ecophysiological traits of *C. edulis*, which includes a facultative C3-CAM physiology (Campoy et al., 2018), explains its tolerance to a wide range of ecological conditions and could contribute to understand how the species can display successful phenotypic responses to our climatic treatments without epigenetic regulation. Thereby, this result reinforces the current knowledge that the magnitude of the epigenetic effects depends on several factors, including the specific environmental stress, the exposure time of plants to stress, or the species under consideration (e.g., Verhoeven et al., 2010; Nicotra et al., 2015; Huang et al., 2017).

### Phenotypic and Epigenetic Differences Between Native and Invasive *Carpobrotus edulis*

Given that the environmental changes faced by invasive plants may be much greater and/or more rapid than those experienced by species under natural conditions, it has been suggested that adaptive plasticity and epigenetic variation may be important mechanisms by which invasive populations can successfully adapt to these novel environments (Richards et al., 2006; Estoup et al., 2016; Mounger et al., 2021).

In this study, we found a divergence between South African and Iberian Peninsula phenotypes and epigenotypes during the process of invasion, regardless of climatic treatments. As inferred from the PCA, the region of origin influenced phenotypic traits (PC 2 = 35.1% of the total variance) of *C. edulis* related to carbon isotope discrimination and biomass partitioning (i.e., on the Δ^13^C and RSR), with plants from the invaded region showing lower values than plants from the native region (Table 2; Figure 1C; Supplementary Table 4). The positive linear relationship between carbon isotopic discrimination and water use efficiency (WUE; Farquhar et al., 1989) indicates a higher efficiency for the use of water in the Iberian Peninsula plants (i.e., lower Δ^13^C values) than in the South African plants, which is consistent with the relatively lower below-ground biomass allocation (i.e., lower RSRs ratios) observed in the invasive Iberian Peninsula plants. Because shifts in these morphological and physiological traits may lead to invasive plants to use water and light more efficiently, this phenotypic divergence can be considered as an adaptive strategy for successful expansion in the introduce range. Other studies have demonstrated rapid genetic shifts in important ecological traits between native and invasive populations after introduction in new territories (Zou et al., 2007; Caño et al., 2008; Matesanz et al., 2012; Roiloa et al., 2016; Portela et al., 2019; Campoy et al., 2021), and also suggest that intraspecific variability in relevant functional traits may play a crucial role in plant invasion. Moreover, another interesting finding of this work is that some phenotypic traits (i.e., PC 3 mainly related to δ^15^N) of *C. edulis* differs between regions of origin, partly through its effect on methylation (Figure 3). This indicates that global DNA methylation may also provide an additional source of intraspecific variation in leaf δ^15^N of *C. edulis* that could significantly affect the N dynamics of invaded coastal ecosystems.

One of the most important findings of this study is that in addition to the phenotypic differentiation between plants from the different regions, we provide evidence for epigenetic differences between native and invasive *C. edulis*, with the invasive individuals from the Iberian Peninsula showing higher levels of global DNA methylation compared to their native counterparts from South Africa (Table 1; Figure 1A). This significantly higher level of methylation in invaders than in natives suggests that the invasion process may have selected plants with a greater capacity for epigenetic control. Previous studies have documented higher epigenetic variation levels in introduced compared to native populations for several species (e.g., Spens and Douhovnikoff, 2016), and changes in DNA methylation of some clonal plants with exposure to different habitat types (Richards et al., 2012) and with climate of origin (Zhang et al., 2016), what suggests that epigenetically regulated phenotypic variation may be crucial for the establishment, spread and invasion success of an invasive population, especially in the absence or with low levels of genetic variation (Mounger et al., 2021). In fact, recent literature has highlighted the potential importance of epigenetic variation for the success of clonal invaders based on two reasons. First, because these processes may provide a non-genetic source of heritable variation that, when related to individual fitness, can contribute to generate adaptive phenotypes through rapid selective changes. Second, because clonal reproduction does not reset epigenetic effects that is thought to occur through meiosis (Verhoeven and Preite, 2014; Mounger et al., 2021). Thus, epigenetic changes in asexual organism could be more stably inherited to the progeny (although they can be also inherited through sexual reproduction as well, see for example Sobral et al., 2021a).

The epigenetic differences that we observed in global DNA methylation between native and invasive plants and the relationship between epigenetic features and phenotypic traits of *C. edulis*, also suggest that epigenetic modifications may be a source of intraspecific functional diversity in this clonal plant, and it may also contribute to the successful and rapid adaptation of this species to new habitats. However, we cannot exclude other factors (i.e., multiple introductions from different source populations and the combination of different reproductive strategies, including hybridization; reviewed by Campoy et al., 2018) that could also contribute to explain the epigenetic differences observed. Thereby, further investigations are needed to unravel the relative importance of genetic vs. epigenetic variation in determining the ecological and evolutionary consequences of invasion.

## Conclusion

We found epigenetic (i.e., global DNA methylation) and phenotypic differences (i.e., biomass partitioning pattern and water take up and use) between individuals from the native (South Africa) and the invaded area (Iberian Peninsula) of *C. edulis*. This divergence between native and invasive populations evidences an intraspecific functional variation during the process of invasion and suggests that phenotypic plasticity and global DNA methylation may be related to the successful and rapid adaptation of this species to new habitats. Moreover, our findings strongly suggest that the fractionating processes in the N cycle might be affected by the changing conditions in temperature and rainfall, by methylation and by region of origin, which in turn could impact the N dynamics in coastal ecosystems invaded by *C. edulis*.

With this work, we also showed that the new climate scenarios projected for Southern Europe (i.e., increased temperature and reduced rainfall) might foster rapid changes in functional traits of *C. edulis,* but, interestingly, we also demonstrated that these phenotypic changes seem to be independent of epigenetic ones. Finally, this study highlights that phenotypic plasticity might improve species fitness in new climatic scenarios and adds to the current evidence of the important role of epigenetic mechanisms for the adaptive success of invasive species to new areas.

## Data Availability Statement

The original contributions presented in the study are included in the article/Supplementary Material. Further inquiries can be directed to the corresponding author.

## Author Contributions

RR designed and conceived the work and together with RB and MS get fundings for the study. RR, JC, and ML conducted field sampling, set up the experiment, and collected data. RB, BC, and JC carried out the multistage study to select primer combinations for the MSAP analyses. MS provided the statistical approach and contributed substantially to analyze data and writing the manuscript. JC led the analysis, manuscript writing, and submission. All authors commented on and reviewed the final draft. All authors contributed to the article and approved the submitted version.

## Funding

Funding for this study was provided by the Spanish Ministry of Economy and Competitiveness and the European Regional Development Fund (ERDF; grant Ref. CGL2013-48885-C2-2-R and Ref. CGL2017-87294-C3-1P awarded to RR) and by the Autonomous Government of Galicia (grant ref. I2CB awarded to MS).

## Conflict of Interest

The authors declare that the research was conducted in the absence of any commercial or financial relationships that could be construed as a potential conflict of interest.

## Publisher’s Note

All claims expressed in this article are solely those of the authors and do not necessarily represent those of their affiliated organizations, or those of the publisher, the editors and the reviewers. Any product that may be evaluated in this article, or claim that may be made by its manufacturer, is not guaranteed or endorsed by the publisher.

## Footnotes

^1^ www.meteogalicia.es
