# Supplementary material for: Epigenetic and Phenotypic Responses to Experimental Climate Change of Native and Invasive Carpobrotus edulis
Source: Front Plant Sci. 2022 Jun 17;13:888391. doi: 10.3389/fpls.2022.888391 (PMC9247612; doi:10.3389/fpls.2022.888391)
Supplement: Supplementary file 1 [file Data_Sheet_1.pdf]

frontiers

Supplementary Material

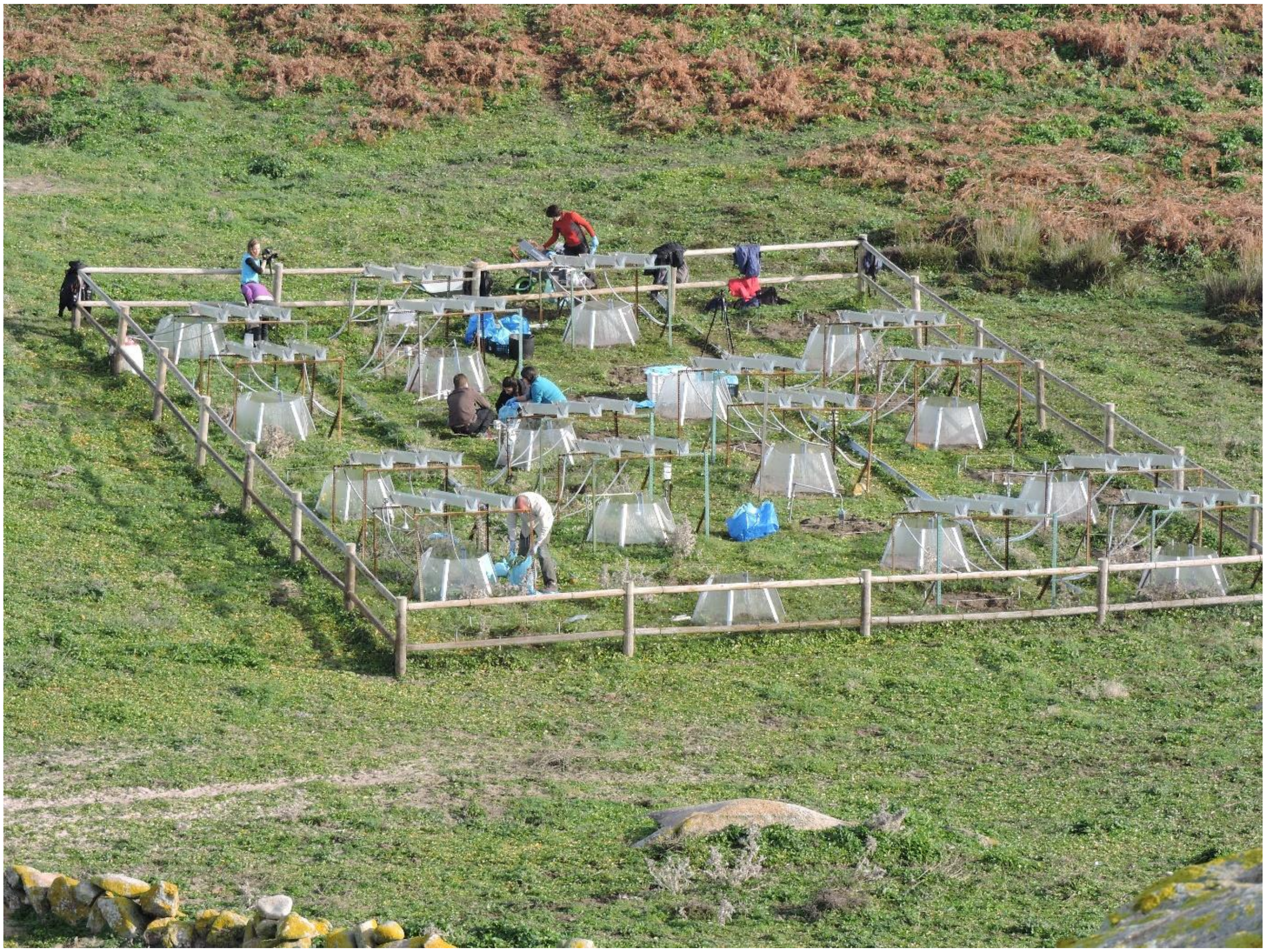

Supplementary Figure 1. General view of the experimental climate change plot in the NE of Sálvora island (42°28′44″N, 9°0′34″W), within the Atlantic Islands of Galicia National Park (northwest Iberian Peninsula).

(A)

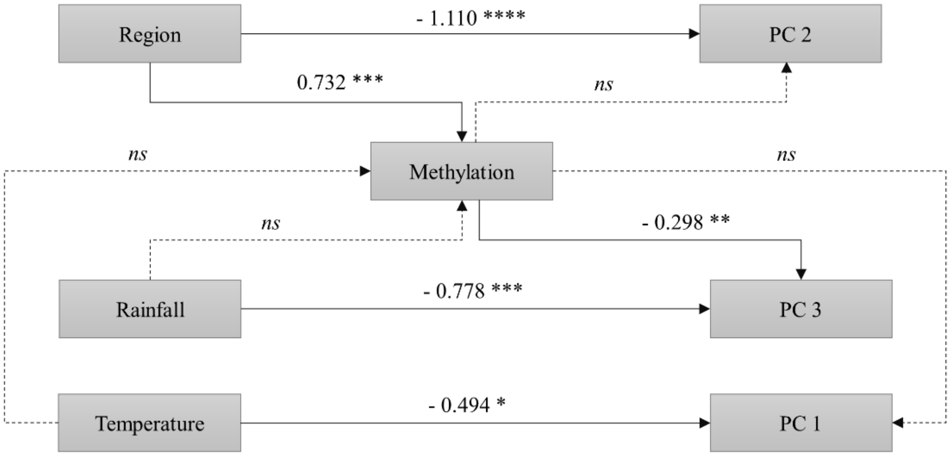

(B)

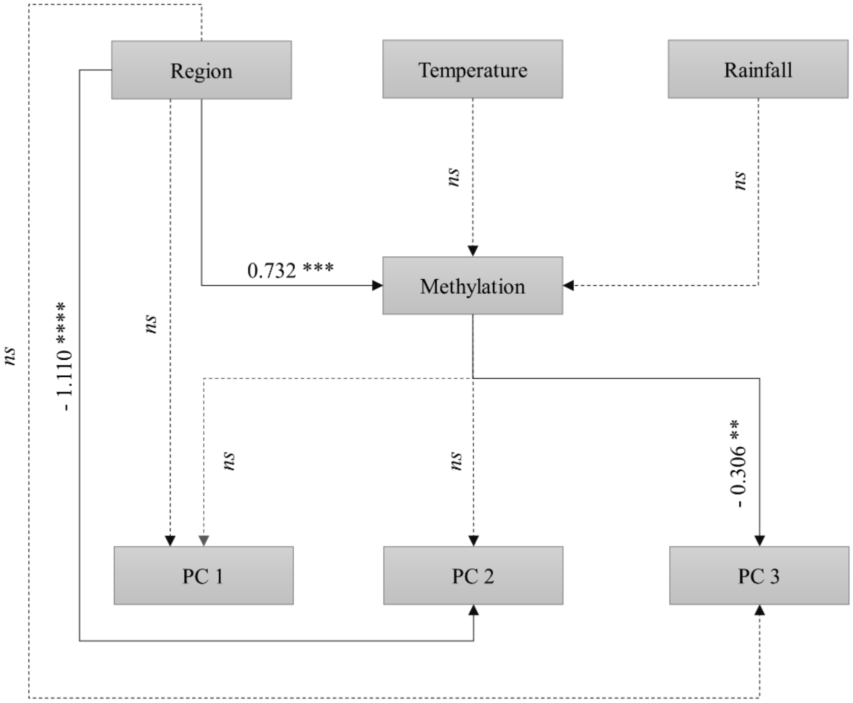

Supplementary Figure 2. Results of alternative hypotheses tested by SEM. We show coefficients for each significant path (*P < 0.1, **P < 0.05, ***P < 0.01, ****P < 0.001). For visualization purposes the strength of non-significant relationships (*ns*; dashes lines) are not shown. (A) shows the initial solution for the definitive model for comparison with alternative hypotheses (B). Models properly fitted the data as shown by the non-significant Chi-squares values (**A**, $\chi 2$: 4.015; df: 12; p: 0.983; **B**, χ^2: 15.002; df: 12; p= 0.241).

(A)

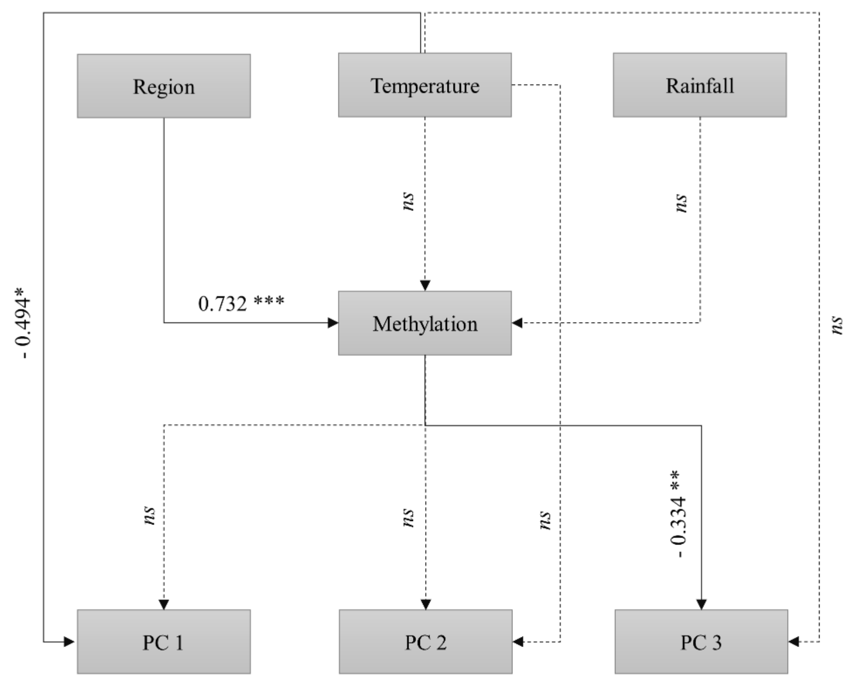

(B)

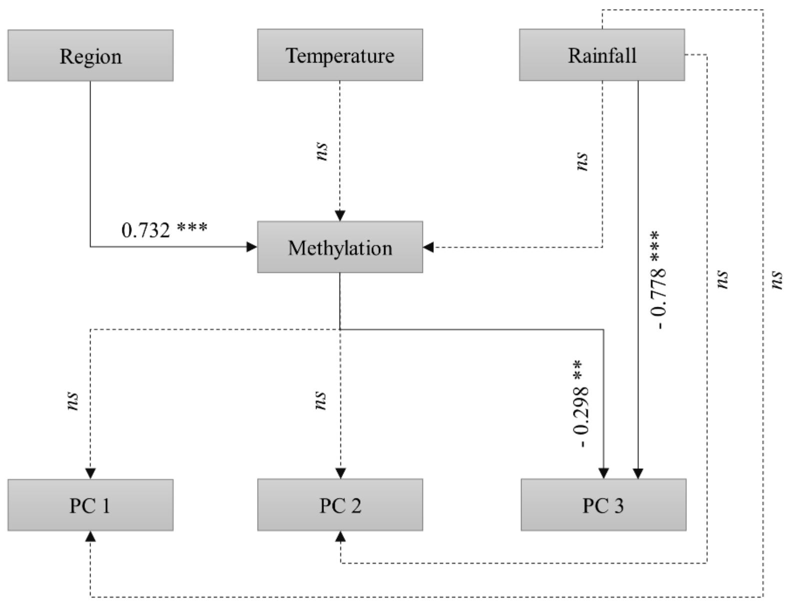

Supplementary Figure 3. Results of alternative hypotheses tested by SEM that did not fit the data. We show coefficients for each significant path (**P* < 0.1, ***P* < 0.05, ****P* < 0.01, *****P* < 0.001). For visualization purposes the strength of non-significant relationships (*ns*; dashes lines) are not shown. Models did not properly fit the data, as shown by the significant Chi-squares values (**A**, χ^2: 27.367; df: 12; p= 0.007; B, χ^2: 21.949; df: 12; p= 0.038).

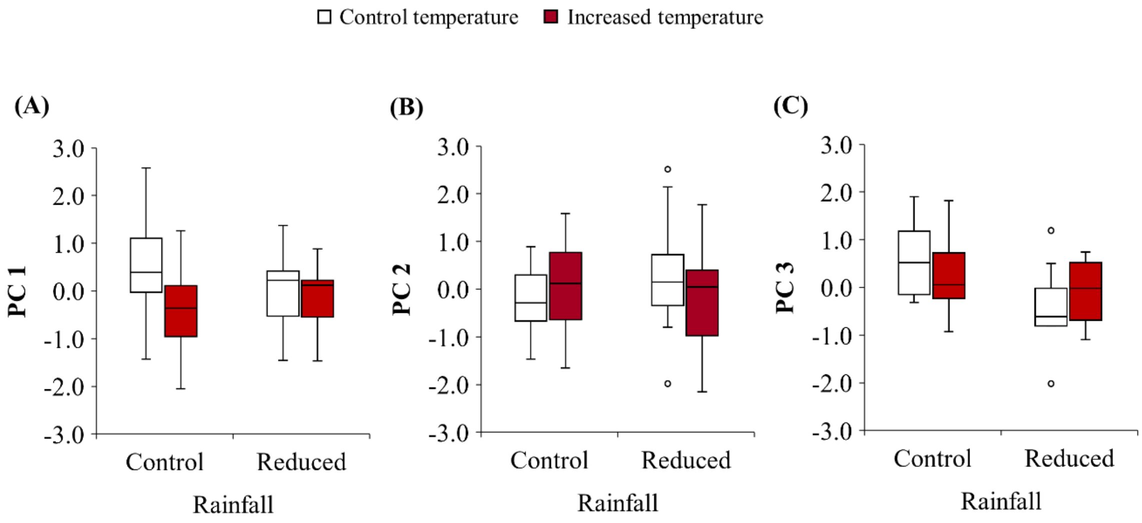

Supplementary Figure 4. Boxplots showing the distribution of data (median, interquartile range, minimum, maximum and the outliers; n = 12) for the combined effects of temperature and rainfall on the first principal component (PC 1 = 35.1% variance) (A), the second principal component (PC 2 = 21.2% variance) (B), and the third principal component (PC 3 = 14.5 % variance) (C) of *C. edulis* phenotype ($F_{1, 36}$ = 3.892; P = 0.056 for the interaction T x R on PC 3).

Supplementary Table 1. Site location of *Carpobrotus edulis* sampled from the invaded region in South Europe (1: Viveiro, Spain; 2: Caminha, Portugal; 3: Castelo do Neiva, Portugal; 4: Quiaios, Portugal) and from the native region in South Africa (5: Cape of Good Hope; 6: Fish Hoek; 7: Kleinmond; 8: Hawston).

Site	Latitude	Longitude
1	43°41'N	7°36'W
2	41°51'N	8°51'W
3	41°37'N	8°48'W
4	40°13'N	8°53'W
5	34°20'S	18°27'E
6	34°07'S	18°26'E
7	34°20'S	19°02'E
8	34°23'S	19°07'E

Supplementary Table 2. Adaptor and primer sequences used for MSAP reactions.

**Reaction**	**Reactant**	**Related endonuclease**	**Sequence 5'-3'**
Ligation	Adaptors	*Eco*RI-adapter top	CTCGTAGACTGCGTACC
		*Eco*RI-adapter bottom	AATTGGTACGCAGTCTAC
		*Hpa*II/*Msp*I-adapter top	GACGATGAGTCTCGAT
		*Hpa*II/*Msp*I-adapter bottom	CGATCGAGACTCAT
Pre-amplification	Pre-selective primers	*Eco*RI-0	GACTGCGTACCAATTC-0
		*Hpa*II/*Msp*I-0	GATGAGTCTCGATCGG-0
Amplification	Selective primers	*Eco*RI-AA/AT/TA/TC/TG	GACTGCGTACCAATTC-AA/AT/TA/TC/TG
		*Hpa*II/*Msp*I-AA/AC/AT	GATGAGTCTCGATCGG-AA/AC/AT

Supplementary Table 3. Primer combinations used for the MSAP selective amplification, number of fragments (loci) generated and scoring error rates.

**Primer combination**	***Eco*RI-primer (5'-3')**	***Hpa*II/*Msp*I-primer (5'-3')**	**Total number of fragments amplified**	**Scoring error rate**
1	GACTGCGTACCAATTC + AA	GAT GAG TCT CGA TCG G + AC	36	4.28
2	GACTGCGTACCAATTC + AT	GAT GAG TCT CGA TCG G + AA	28	2.83
3	GACTGCGTACCAATTC + AT	GAT GAG TCT CGA TCG G + AC	36	4.92
4	GACTGCGTACCAATTC + TA	GAT GAG TCT CGA TCG G + AA	49	3.66
5	GACTGCGTACCAATTC + TC	GAT GAG TCT CGA TCG G + AT	38	3.70
6	GACTGCGTACCAATTC + TG	GAT GAG TCT CGA TCG G + AC	36	3.96
Total			**223**	**3.89**

Supplementary Table 4. Variation explained and trait loadings of principal components (PC 1, PC 2 and PC 3) in the PCA performed on seven phenotypic traits (relative growth rate, RGR [$year^{-1}$]; root to shoot ratio, RSR; leaf carbon and nitrogen ratio, C/N; carbon isotopic discrimination, $\Delta^{13}C$ [‰]; nitrogen isotopic composition, $\delta^{15}N$ [‰]; Structural Independent Pigment Index, SIPI, and the Photochemical Reflectance Index, PRI_{531}) of *Carpobrotus edulis* plants from the native and the invaded region grown under two temperatures (control vs. increased), and two rainfall levels (control vs. reduced) throughout 14 months. The variables with the highest loads for each principal component are highlighted in bold.

	**PC 1**	**PC 2**	**PC 3**
Variation explained (%)	**35.1**	**21.2**	**14.5**
Variable loadings			
PRI_{531}	**-0.870**	-0.218	0.023
SIPI	**0.784**	0.287	-0.003
RGR	**-0.707**	0.139	0.490
C/N	**0.617**	-0.484	-0.009
$\Delta^{13}C$ (‰)	0.008	**0.836**	-0.188
RSR	0.040	**0.622**	0.289
$\delta^{15}N$ (‰)	0.452	-0.110	**0.807**
